# Melanocyte and Melanoma Cell Activation by Calprotectin

**DOI:** 10.1155/2014/846249

**Published:** 2014-08-12

**Authors:** Stephanie H. Shirley, Kristine von Maltzan, Paige O. Robbins, Donna F. Kusewitt

**Affiliations:** Department of Molecular Carcinogenesis, Science Park, University of Texas MD Anderson Cancer Center, 1808 Park Road 1C, Smithville, TX 78957, USA

## Abstract

Calprotectin, a heterodimer of S100A8 and S100A9, is a proinflammatory cytokine released from ultraviolet radiation-exposed keratinocytes. Calprotectin binds to Toll-like receptor 4, the receptor for advanced glycation end-products, and extracellular matrix metalloproteinase inducer on target cells to stimulate migration. Melanocytes and melanoma cells produce little if any calprotectin, but they do express receptors for the cytokine. Thus, keratinocyte-derived calprotectin has the potential to activate melanocytes and melanoma cells within the epidermis in a paracrine manner. We examined the ability of calprotectin to stimulate proliferation and migration in normal human melanocytes and melanoma cells *in vitro*. We first showed, by immunofluorescence and quantitative RT-PCR, that the melanocytic cells employed expressed a calprotectin receptor, the receptor for advanced end-products. We then demonstrated that calprotectin significantly enhanced proliferation, migration, and Matrigel invasion in both normal human melanocytes and melanoma cells. Thus, calprotectin is one of the numerous paracrine factors released by ultraviolet radiation-exposed keratinocytes that may promote melanomagenesis and is a potential target for melanoma prevention or therapy.

## 1. Introduction

In normal human epidermis, each melanocyte associates with approximately 35 keratinocytes to form an “epidermal melanin unit” [1]. The keratinocytes in each unit exert considerable control over the behavior of the associated melanocyte, via interactions of cell-cell adhesion molecules and release of paracrine factors. Ultraviolet radiation (UVR) can substantially alter keratinocyte-melanocyte interactions. UVR exposure enhances keratinocyte production of a wide variety of paracrine factors, including interleukins, growth factors, interferons, and chemokines, that may profoundly affect melanocyte and melanoma cell proliferation, migration, and gene expression [1–4]. Such paracrine modulation of melanocyte behavior may promote melanomagenesis [1, 4].

Within the cell, S100 proteins, in the form of heterodimers or homodimers, control the localization and activity of a variety of target proteins; however, the S100 proteins S100A8 and A9 are also secreted from cells as calprotectin, a heterodimeric proinflammatory cytokine [5]. Calprotectin exerts its effects by binding to a variety of receptors on the surface of target cells, including Toll-like receptor 4 (TLR4), the receptor for advanced glycation end-products (RAGE), and extracellular matrix metalloproteinase inducer (EMMPRIN) [5–7]. S100A8 and A9 are expressed at extremely low levels in unperturbed keratinocytes, but their expression is readily stimulated by a variety of insults, including UVR exposure [8]. Calprotectin is chemotactic for leukocytes and keratinocytes; thus, it stimulates keratinocyte motility and recruits inflammatory cells to the skin [9]. Melanocytes and melanoma cells themselves do not appear to express significant levels of S100A8 and A9 [10], although they do express the calprotectin receptors RAGE, TLR4, and EMMPRIN [7, 11, 12]. Based on these observations, we investigated a possible role for calprotectin in the paracrine activation of melanocytes and melanoma cells.

## 2. Materials and Methods

### 2.1. Cells

Normal human melanocytes (NHM) and normal human keratinocytes of neonatal origin were obtained from the American Type Culture Collection (Manassas, VA) and were maintained in dermal basal medium supplemented with a melanocyte or keratinocyte growth kit (ATCC) as appropriate. The WC62 melanoma cell line (Coriell Institute, Camden, NJ), derived from the primary melanoma of a patient with metastatic disease, was grown in Eagle's modified essential medium (ATCC) supplemented with 10% normal calf serum (Thermo Scientific, Hudson, NJ).

### 2.2. Immunofluorescence

Cells grown on coverslips were fixed briefly in paraformaldehyde and nonspecific binding was blocked with 5% BSA in Tris-buffered saline containing 0.05% Tween. Coverslips were incubated with a rabbit polyclonal anti-RAGE primary antibody (Abcam, Cambridge, MA) diluted in blocking buffer for 2 hours at room temperature and then with Alexa 488-labeled goat anti-rabbit Fab secondary antibody (Life Technologies, Grand Island, NY) for 1 hour in the dark at room temperature. Control slides were incubated with normal goat IgG (R&D Systems, Minneapolis, MN) at a protein concentration equivalent to the primary antibody before addition of secondary antibody. Cells were examined by confocal microscopy.

### 2.3. qRT-PCR

To validate expression of RAGE, mRNA was isolated from normal human keratinocytes, NHM, and WC62 cells using the Qiagen RNeasy kit followed by QIAshredder treatment (Qiagen, Valencia, CA). After reverse transcription and DNAse treatment, RAGE and GAPDH mRNA levels were determined using TaqMan gene expression assays (Hs00542584_g1 and Hs02758991_g1, Life Technologies, Grand Island, NY) as directed together with TaqMan Universal Master Mix (4304437, Applied Biosystems). Amplifications were performed on a 7900HT real-time PCR analyzer (Applied Biosystems). Relative gene expression was calculated using the comparative C(T) method [13], using GAPDH expression to calculate delta CT and determining fold change compared to expression in normal human keratinocytes.

### 2.4. Cell Proliferation Assay

To assay the effect of calprotectin on melanocyte and melanoma cell proliferation, we treated the cells with calprotectin, using recombinant S100A8 and S100A9 (Abnova, Taiwan) allowed to dimerize* in vitro* [14]. Cells were seeded at 1–5 × 10^4^ cells per well in 96-well dishes in standard medium or medium containing calprotectin at a final concentration of 100 pg/mL S100A8 and 1 ng/mL S100A9. On the succeeding 5 days, 3-[4,5-dimethylthiazol-2-yl]-2,5-diphenyl tetrazolium bromide (MTT) (Sigma, St. Louis, MO) dissolved in freshly prepared 0.1 N HCl in anhydrous isopropanol at a concentration of 5 mg/mL was added and cells were incubated for 3 hours. Absorbance was measured at 570 nm.

### 2.5. Cell Migration Assay

To evaluate the effect of calprotectin on migration, cells were plated into uncoated or Matrigel-coated transwell chambers (Becton, Dickinson and Company Biosystems, Bedford, MA). Lower chambers were filled with medium alone or with medium containing recombinant S100A8 and S100A9 [14]. After 24 hours, upper chambers were cleaned of remaining cells and membranes were fixed and stained to visualize migrating cells (DiffQuick kit, Sigma, St. Louis, MO). Dye was extracted from transwell membranes using methanol and absorbance measured with a spectrophotometer at 550 nm. All migration assays were conducted in the presence of 10 *μ*g/mL mitomycin c (Sigma, St. Louis, MO) to block proliferation.

## 3. Results and Discussion

**Figure 1:**
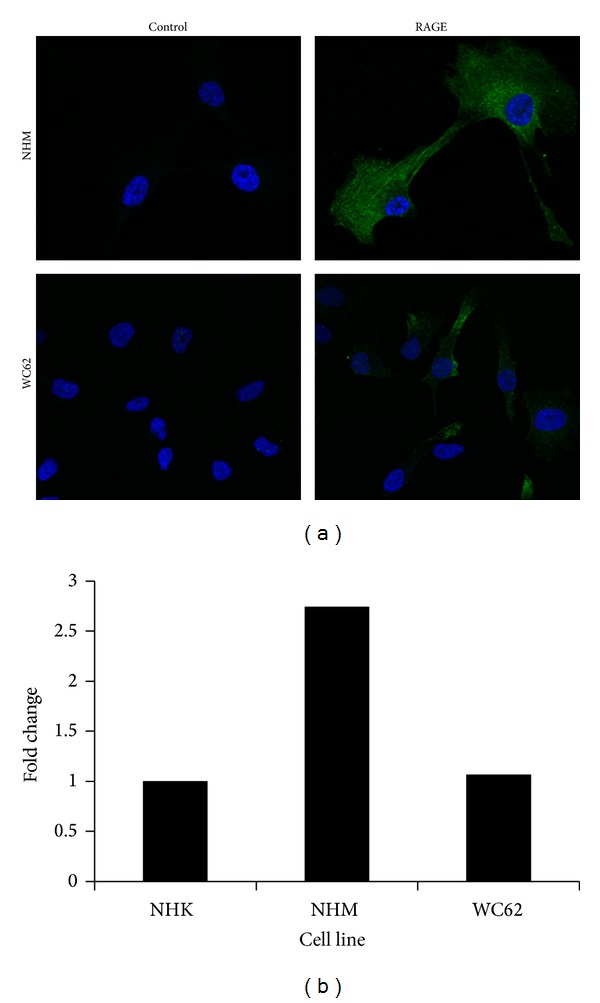
Expression of the calprotectin receptor RAGE on melanocytes and melanoma cells. NHM or WC62 cells were labeled with anti-RAGE primary antibodies followed by fluorescently labeled secondary antibody, as described in Materials and Methods, and examined by confocal microscopy. Control samples were treated with a protein concentration of normal goat IgG equivalent to that of the primary antibody.

As shown in Figure 1(a), both cell types expressed detectable levels of surface RAGE, the canonical calprotectin receptor, in agreement with previous reports [11]. Expression of RAGE mRNA was confirmed by qRT-PCR (Figure 1(b)). Compared to normal human keratinocytes, NHM expressed more than twice the level and WC62 cells expressed a comparable level of RAGE mRNA. Previous studies have demonstrated RAGE expression in cultured normal human keratinocytes [15]. Both immunofluorescence and qRT-PCR results suggest higher expression of RAGE in NHM than in WC62 cells.

**Figure 2:**
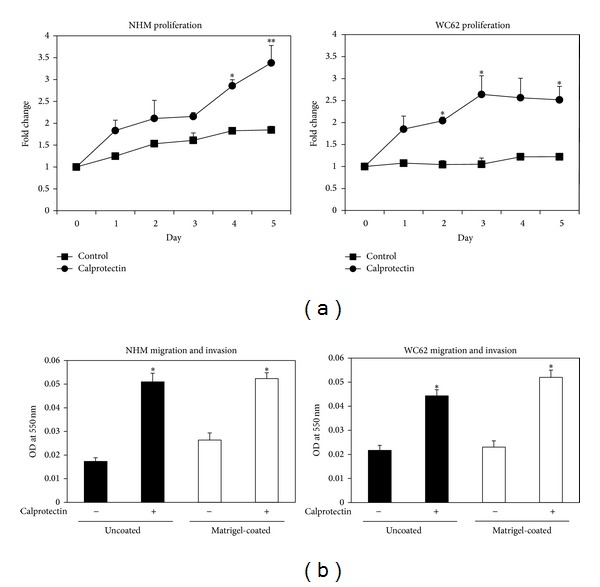
Melanocyte and melanoma cell proliferation and migration in response to calprotectin. (a) NHM or WC62 cells were plated in 96-well plates, treated with a mixture of recombinant S100A8 (100 pg/mL) and S100A9 (1 ng/mL), and allowed to dimerize* in vitro*. Cell number was determined using the MTT assay. Triplicate samples were run for each treatment in each experiment and the experiments were repeated 5 times. Bars indicate SEM. Asterisks indicate a significant difference (*P* < 0.05) between control and calprotectin-treated cells as determined by 2-tailed Student *t*-test assuming unequal variance. (b) NHM or WC62 cells were plated in uncoated or Matrigel-coated transwell chambers containing the appropriate growth medium. Lower chambers were filled with medium alone or with medium containing recombinant S100A8 (100 pg/mL) and S100A9 (1 ng/mL) (Abnova, Taiwan) and were allowed to dimerize* in vitro*. Twenty-four hours later, the number of cells that had migrated through the membrane was determined as described in the text. Three replicate experiments were performed. Bars indicate SD. Asterisks indicate a significant difference (*P* < 0.05) between control and calprotectin-treated cells as determined by 2-tailed Student *t*-test assuming unequal variance.

As shown in Figure 2(a), a significant difference in cell number between control and calprotectin-treated cells appeared at 4 days of treatment in NHM and at 2 days of treatment in WC62 cells. Calprotectin stimulated a maximum increase in cell number of 1.8-fold in NHM and 2.5-fold in WC62 cells compared to control cells. Movement through untreated membranes (migration) and through Matrigel-coated membranes (invasion) was significantly enhanced by 2- to 3-fold in both melanocytes and melanoma cells treated with calprotectin compared to control cells (Figure 2(b)).

It is important to note that these studies do not indicate that RAGE is the calprotectin receptor responsible for the effect of calprotectin on NHM and WC62 melanoma cell proliferation and migration, only that these cells express at least one calprotectin receptor. Another study has shown that overexpression of RAGE in a human melanoma cell line is associated with not only increased migration but also reduced proliferation in contrast with our study, [16]. Other calprotectin receptors, including TLR4 and EMMPRIN, may play important roles in mediating calprotectin effects on cells of melanocytic origin. Indeed, a recent study demonstrates that S100A9, probably also calprotectin, is a ligand for EMMPRIN and that EMMPRIN overexpression enhances and EMMPRIN blockade suppresses the migration of melanoma cell lines in response to S100A9 treatment [7]. Moreover, downregulation of EMMPRIN expression in melanoma cells reduces both proliferation and migration [17]. However, it does not appear that EMMPRIN is expressed at appreciable levels on normal human melanocytes [18].

It is clear from these studies that exogenous calprotectin can activate melanocytes and melanoma cells to proliferate and to migrate. Thus, calprotectin appears to be one of the numerous paracrine factors released by UVR-exposed keratinocytes that may promote melanomagenesis. Blocking the induction, release, or activity of calprotectin may thus represent a potential preventative or therapeutic strategy for melanoma. TLRs and RAGEs, receptors for calprotectin and for a variety of other ligands, are being considered as therapeutic targets for a wide array of diseases, including sepsis, asthma, and diabetes [19, 20]. A number of approaches to blocking signaling through these receptors are under investigation and may prove valuable in preventing or treating melanoma. However, further studies are clearly needed to determine the importance of calprotectin in melanoma development and progression and the therapeutic benefit of blocking its activity.

## 4. Conclusion

Calprotectin is one of many proinflammatory mediators released from UVR-exposed keratinocytes. We have shown that melanocytes and melanoma cells express RAGE, the canonical calprotectin receptor, and that calprotectin stimulates these cells to proliferate and to migrate. Because calprotectin activates melanocytes and melanoma cells, it is a potential target for intervention in melanomagenesis.

## Abbreviations

EMMPRIN: Extracellular matrix metalloproteinase inducer
MTT: 3-[4,5-Dimethylthiazol-2-yl]-2,5-diphenyl tetrazolium bromide
NHM: Normal human melanocytes
RAGE: Receptor for advanced glycation end-products
TLR: Toll-like receptor
UVR: Ultraviolet radiation.

## References

[1] Lee JT, Herlyn M (2007). Microenvironmental influences in melanoma progression. *Journal of Cellular Biochemistry*.

[2] Brenner M, Degitz K, Besch R, Berking C (2005). Differential expression of melanoma-associated growth factors in keratinocytes and fibroblasts by ultraviolet A and ultraviolet B radiation. *British Journal of Dermatology*.

[3] Imokawa G (2004). Autocrine and paracrine regulation of melanocytes in human skin and in pigmentary disorders. *Pigment Cell Research*.

[4] Richmond A, Yang J, Su Y (2009). The good and the bad of chemokines/chemokine receptors in melanoma. *Pigment Cell *and* Melanoma Research*.

[5] Ehrchen JM, Sunderkötter C, Foell D, Vogl T, Roth J (2009). The endogenous Toll-like receptor 4 agonist S100A8/S100A9 (calprotectin) as innate amplifier of infection, autoimmunity, and cancer. *Journal of Leukocyte Biology*.

[6] Donato R (2001). S100: a multigenic family of calcium-modulated proteins of the EF-hand type with intracellular and extracellular functional roles. *International Journal of Biochemistry and Cell Biology*.

[7] Hibino T, Sakaguchi M, Miyamoto S (2013). S100A9 is a novel ligand of EMMPRIN that promotes melanoma metastasis. *Cancer Research*.

[8] Marionnet C, Bernerd F, Dumas A (2003). Modulation of gene expression induced in human epidermis by environmental stress in vivo. *Journal of Investigative Dermatology*.

[9] Eckert RL, Broome A, Ruse M, Robinson N, Ryan D, Lee K (2004). S100 proteins in the epidermis. *Journal of Investigative Dermatology*.

[10] Petersson S, Shubbar E, Enerbäck L, Enerbäck C (2009). Expression patterns of S100 proteins in melanocytes and melanocytic lesions. *Melanoma Research*.

[11] Leclerc E, Heizmann CW, Vetter SW (2009). RAGE and S100 protein transcription levels are highly variable in human melanoma tumors and cells. *General Physiology and Biophysics*.

[12] Ahn JH, Park TJ, Jin SH, Kang HY (2008). Human melanocytes express functional toll-like receptor 4. *Experimental Dermatology*.

[13] Schmittgen TD, Livak KJ (2008). Analyzing real-time PCR data by the comparative CT method. *Nature Protocols*.

[14] Saha A, Lee Y, Zhang Z, Chandra G, Su S, Mukherjee AB (2010). Lack of an endogenous anti-inflammatory protein in mice enhances colonization of B16F10 melanoma cells in the lungs. *The Journal of Biological Chemistry*.

[15] Djerbi N, Dziunycz PJ, Reinhardt D (2013). Influence of cyclosporin and prednisolone on RAGE, S100A8/A9, and NF*κ*B expression in human keratinocytes. *JAMA Dermatology*.

[16] Meghnani V, Vetter SW, Leclerc E (2014). RAGE overexpression confers a metastatic phenotype to the WM115 human primary melanoma cell line. *Biochimica Biophysica Acta*.

[17] Chen X, Lin J, Kanekura T (2006). A small interfering CD147-targeting RNA inhibited the proliferation, invasiveness, and metastatic activity of malignant melanoma. *Cancer Research*.

[18] Su J, Chen X, Kanekura T (2009). A CD147-targeting siRNA inhibits the proliferation, invasiveness, and VEGF production of human malignant melanoma cells by down-regulating glycolysis. *Cancer Letters*.

[19] Yamagishi S, Nakamura K, Matsui T, Ueda S, Fukami K, Okuda S (2008). Agent that block advanced glycation end product (AGE)-RAGE (receptor for AGEs)-oxidative stress system: a novel therapeutic strategy for diabetic vascular complications. *Expert Opinion on Investigational Drugs*.

[20] Zuany-Amorim C, Hastewell J, Walker C (2002). Toll-like receptors as potential therapeutic targets for multiple diseases. *Nature Reviews Drug Discovery*.

